# Supporting community‐dwelling older people with cognitive impairment to stay at home: A modelled cost analysis

**DOI:** 10.1111/ajag.12818

**Published:** 2020-07-01

**Authors:** Suzanne M. Dyer, Lachlan B. Standfield, Nicola Fairhall, Ian D. Cameron, Meredith Gresham, Henry Brodaty, Maria Crotty

**Affiliations:** ^1^ Rehabilitation, Aged and Extended Care College of Medicine and Public Health Flinders University Adelaide South Australia Australia; ^2^ Threshold Economics Ltd Auckland New Zealand; ^3^ Institute for Musculoskeletal Health The University of Sydney Sydney New South Wales Australia; ^4^ John Walsh Centre for Rehabilitation Research Faculty of Medicine and Health The University of Sydney Sydney New South Wales Australia; ^5^ Dementia Centre for Research Collaboration School of Psychiatry University of NSW Sydney New South Wales Australia; ^6^ The Dementia Centre Hammond Care Sydney New South Wales Australia; ^7^ Centre for Healthy Brain Ageing (CHeBA) School of Psychiatry University of NSW Sydney New South Wales Australia

**Keywords:** caregivers, dementia, home care, nursing homes, health resource

## Abstract

**Objective:**

To model the potential financial implications of Australian programs supporting cognitively impaired community‐dwelling older people.

**Methods:**

Markov cohort models of (a) an observational study of a residential dyadic training program for carers and people with dementia (GTSAH) and (b) a frailty intervention (FIT) in a cognitively impaired subgroup. Direct health and social welfare costs accrued over 5 years (2018 $AUD prices) were captured. GTSAH costs $3755, FIT costs $1834, and permanent residential aged care (P‐RAC) costs $237 per day.

**Results:**

Modelling predicted costs break even in approximately 5 months for GTSAH and 7 months for FIT, after which these interventions saved funds. The primary driver of savings was the P‐RAC cost (discounted at 5%/annum), at $121 030 for GTSAH vs $231 193 for standard care; and $47 857 with FIT vs $111 359 for standard care.

**Conclusions:**

Programs supporting cognitively impaired community‐dwelling older people could be financially beneficial; further evaluation and implementation would be a worthwhile investment.

**Policy Impact**

Government investment in the implementation and further evaluation of programs that support older, frail and cognitively impaired people to remain at home for longer supports the wishes of older Australians and is a low‐risk financial investment, with potential for large savings to government from delayed admission to permanent residential care.

**Practice Impact**

Health and aged care practitioners and organisations should be encouraged to provide structured evidence‐based programs such as the FIT and GTSAH interventions to support cognitively impaired community‐dwelling older people to remain living at home.

## 1 INTRODUCTION

The World Health Organization has named dementia as a public health priority and governments worldwide need to prepare for the increase in support required in coming years.^1^, ^2^ Most people want to remain living in their own homes, and the costs of providing permanent residential aged care (P‐RAC) for people living with dementia may not be sustainable.^3^ Internationally, the focus on providing services to support older people to remain living at home has increased. For example, in Denmark most long‐term care is provided at home, with a strong emphasis on rehabilitative approaches.^4^, ^5^ In Australia, government programs provide community home support (including home modifications and limited rehabilitation), home care packages (HCP including domestic duties, personal care and limited nursing services), respite programs and transition care.^6^

A recent meta‐analysis found that multicomponent carer interventions reduce the risk of institutionalisation of people living with dementia.^7^ Carers of people living with dementia can also realise benefits in terms of reductions in depression, functional decline and carer burden.^7^, ^8^ Carer distress and relationships are also predictors of admission to P‐RAC for home care recipients.^9^

The whole of system (health and social care) annual cost of providing P‐RAC for a person living with dementia in Australia has recently been estimated as $AUD88 000 (2016 prices).^10^ This estimate enables modelling of the potential financial implications of providing additional services to support people living with dementia in the community, capturing potential delays in admission to P‐RAC.

This study modelled the longer‐term costs associated with providing two Australian programs to support people with dementia or cognitive impairment living in the community in comparison with standard care (SC, including home care packages, standard medical services and respite). We hypothesised that the costs of providing these programs could be offset by delays in P‐RAC admissions.

## 2 METHODS

### 2.1 Modelled programs

The approach to selecting Australian community‐based programs with some evidence for supporting older people to remain living in the community is described in the Supporting Information. Two programs were selected for modelling.

An Australian randomised controlled trial (RCT) examined the effectiveness of a dyadic residential support program for people living with dementia and their carers. The program comprised a 10‐day inpatient hospital carer training program for carers and a memory training and activity program for the people living with dementia, with follow‐up and phone support over 12 months.^11^ Follow‐up to 8 years postintervention found delayed admission to P‐RAC and increased survival for those living with dementia.^11^ Economic analysis reported cost savings in comparison with standard respite over the first 3 years.^12^ This intervention has been subsequently implemented as a shorter program delivered over 5 days in a RAC setting (the Going to Stay at Home program; GTSAH). Rates of permanent admissions for participants living with dementia in this program were lower than in a non‐randomised comparison group of people receiving standard residential respite, after 12 months (probability P‐RAC, admission per month GTSAH 0.02 vs standard care 0.05; odds ratio 5.8, 95% confidence interval 2.8‐11.6, *P* < 0.001, n = 85).^13^

The Frailty Intervention Trial (FIT) was an Australian RCT of a 12‐month interdisciplinary, multifactorial rehabilitation intervention.^14^ It provided specialist rehabilitation physician or geriatrician management, physiotherapy, dietitians, mobility aids and other services as required.^15^ After the intervention, the prevalence of frailty in 241 community‐dwelling people aged 70 or older was significantly reduced. The cost of transitioning someone out of frailty was $AUD15, 955 (2011 prices); FIT was reported to be cost‐effective. The trial also reported non‐significant reductions in costs for high‐ and low‐level P‐RAC with the FIT intervention. However, detailed analysis of this outcome has not been previously conducted.^16^ A subgroup analysis was conducted for the subgroup of the 48 cognitively impaired (MMSE 18‐24) participants in this trial, to provide data for the population used in this modelled analysis. Baseline characteristics and admission to P‐RAC data in the subgroup were examined.

### 2.2 Markov models

Two Markov models were developed to compare the costs accrued in Australian community‐based cohorts of people living with dementia or cognitive impairment. The first model (Model 1) compared the GTSAH dyad program with SC. The second model (Model 2) compared the FIT intervention with SC. The models used a 1‐month cycle length with a time horizon of 5 years. The models assumed continuation of observed study effects to 5 years, with sensitivity analyses exploring the impact of this assumption. The models included a broad array of direct health and social welfare costs, most of which are borne by the Australian Government. The models were constructed in Microsoft Excel 2016. In the base case, a discount rate of 5% per annum was applied to adjust costs and benefits realised in the future to the net present value to account for time preference (ie, that people prefer to realise benefits sooner but incur costs later), risk, opportunity cost and diminishing marginal utility. This procedure is in line with recommendations from the Australian Pharmaceutical Benefits Advisory Committee.^17^ All costs are reported in Australian dollars (AUD) at 2018 prices. Half‐cycle correction was applied in the model, as appropriate. The analysis did not capture productivity costs.

**FIGURE 1:**
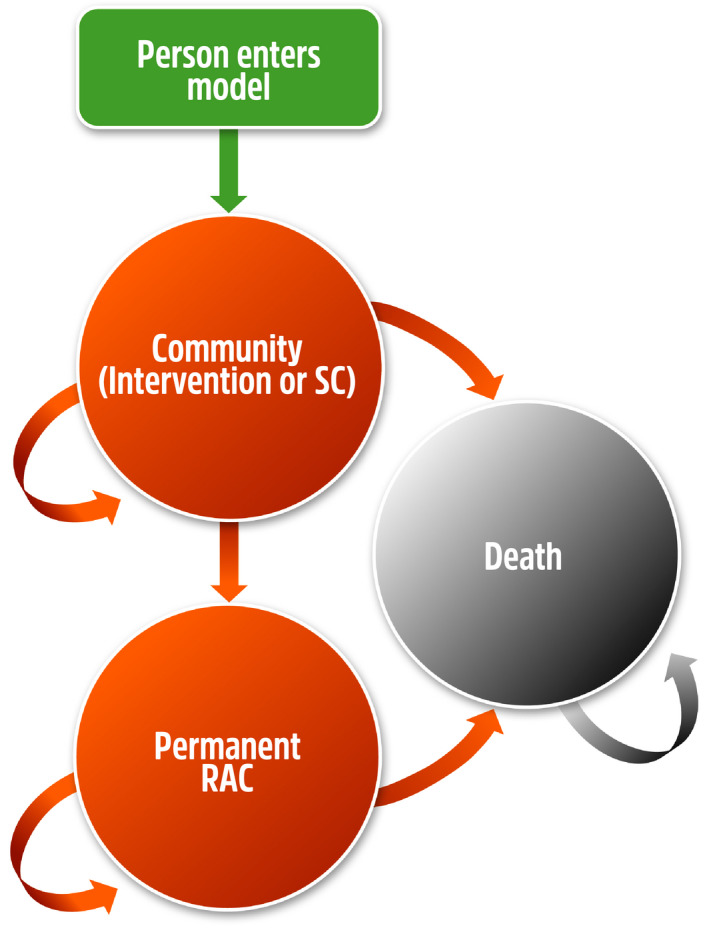
Simplified model structure

Both models had the same structure, consisting of three health states: (a) *Community*; (b) Permanent RAC (*P‐RAC*); and (c) *Death* (Figure 1). The modelled cohort initially entered the model in the *Community* health state. While residing in this health state, the cohort accrued the costs of the program (either GTSAH or FIT in the intervention arm of the models only), hospitalisation, respite, HCP, general practitioner (GP) and non‐medical health practitioner visits. From this health state, the cohort could transition to the *P‐RAC* or *Death* health states. In the *P‐RAC* health state, the cohorts accrued the costs of providing P‐RAC, hospitalisation, clinician and other attendances; diagnostic and pathology services; and pharmaceuticals. From this health state, the cohort could only transition to the *Death* health state (an absorbing health state) in which no costs are accrued.

### 2.3 Model input parameters

Each model had the same structure, with distinct input parameters reflecting the interventions and target populations of interest (for details, see Supporting Information Tables S1 and S2).

Briefly, for Model 1, the cost of the GTSAH intervention reflected the cost of providing the program to a single person with dementia and their carer (ie, a dyad) when run concurrently for a group of four dyads (Email M Gresham to S Dyer 20 Sept 2018). The intensity of resource use in the community setting (eg, GP visits, allied health use, residential respite and hospitalisation from the community), cohort age and the probability of entry into P‐RAC were sourced from publications of the GTSAH program for the GTSAH and SC arms of the model, with 2018 unit costs applied.^11^, ^12^, ^13^ The probability of admission to P‐RAC was 0.05 per month in the standard care arm and 0.02 in the intervention.^13^

For Model 2, the cost of the FIT intervention was obtained from the RCT.^14^ The intensity of resource use in the community setting (eg, GP visits, transport, home help, meal delivery, allied health use, respite care and hospitalisation), cohort age and the probability of entry into P‐RAC were sourced from a subgroup analysis of the RCT for the FIT and SC arms of the model (participants with a Mini‐Mental State Examination, MMSE, of 18‐24).^14^ While the original FIT differentiated between high‐ and low‐level P‐RAC, probabilities of admission to any level of P‐RAC were used in this analysis as this distinction is no longer used in the Australian aged care system. Thus, costs applied were for P‐RAC broadly.

In both models, the probability of community aged care package use by people with dementia living in the community health state was assumed to be the same for the intervention and SC model arms, based on a similar population of people with dementia who had received an aged care assessment in South Australia (from the Registry of Senior Australians, ROSA). Similarly, the cost of P‐RAC, hospitalisation from P‐RAC and other costs for P‐RAC residents were assumed to be the same in the cohort in the P‐RAC health state for both intervention and SC arms of the model and were derived from an Australian study.^10^ Mortality for people with dementia was derived from relative risk estimates reported by Knopman et al and actuarial life tables from the ABS and was assumed to be identical across model arms.^18^, ^19^

Service use in the standard care arm of the models was based on the published evaluations and included the use of home care packages and standard medical services and respite.^12^, ^13^

### 2.4 Sensitivity analyses

Univariate and multivariate sensitivity analyses were undertaken to explore the impact of altering the input parameters on the modelled findings (Table 1). These included altering the modelled time horizon (ranging from the end of the follow‐up period of the studies at 12 months, to 7 years), discount rates, probability of admission to P‐RAC, cohort age, dementia‐specific excess mortality and combinations of these factors, halving or doubling intervention costs, hospitalisation costs and costs of P‐RAC or respite RAC.

**TABLE 1:** Total cumulative cost (2018 $AUD) of Going to Stay at Home (GTSAH) or Frailty Intervention Trial (FIT) interventions vs standard care (SC): univariate and multivariate sensitivity analyses

Analysis description	Modelled GTSAH cohort costs			Modelled FIT cohort costs		
	Intervention arm	SC arm	Incremental cost	Intervention arm	SC arm	Incremental cost
Base case	$163 716	$270 035	−$106 319	$117 201	$161 350	−$44 149
Time horizon ↓ to 1 y	$21 553	$32 801	−$11 248	$30 385	$34 031	−$3646
Time horizon ↓ to 3 y	$84 511	$147 537	−$63 026	$81 592	$108 617	−$27 025
Time horizon ↑ to 7 y	$244 113	$378 287	−$134 174	$137 105	$189 311	−$52 206
Discount rate ↓ to 0%	$188 110	$309 441	−$121 331	$130 588	$180 935	−$50 347
Discount rate ↑ to 10%	$144 026	$238 082	−$94 056	$106 179	$145 245	−$39 067
Intervention costs halved	$161 839	$270 035	−$108 197	$116 284	$161 350	−$45 066
Intervention costs doubled	$167 471	$270 035	−$102 564	$119 034	$161 350	−$42 316
Hospital costs halved	$155 859	$259 796	−$103 937	$96 742	$146 404	−$49 662
Hospital costs doubled	$179 431	$290 514	−$111 083	$158 118	$191 242	−$33 124
P‐RAC costs halved	$103 201	$154 439	−$51 237	$93 272	$105 671	−$12 399
P‐RAC costs doubled	$284 746	$501 229	−$216 483	$165 058	$272 709	−$107 651
Respite RAC costs halved	$162 830	$269 753	−$106 922	$117 071	$160 816	−$43 744
Respite RAC costs doubled	$165 487	$270 600	−$105 113	$117 460	$162 419	−$44 960
Prob of receiving HCP doubled	$179 923	$278 653	−$98 730	$130 666	$170 441	−$39 775
Prob of receiving a HCP halved	$155 613	$265 726	−$110 114	$110 468	$156 805	−$46 337
Difference in prob of entry to P‐RAC between int and SC arms halved	$228 245	$270 035	−$41 790	$144 447	$161 350	−$16 903
Difference in prob of entry to P‐RAC between int and SC arms doubled	$163 716	$309 976	−$146 259	$117 201	$187 976	−$70 775
Cohort age ↓ by a decade	$172 579	$284 366	−$111 787	$167 467	$235 042	−$67 575
Cohort age ↑ by a decade	$135 960	$225 024	−$89 065	$56 818	$73 650	−$16 832
Dementia‐specific excess mortality doubled	$156 765	$258 768	−$102 003	$94 997	$128 921	−$33 925
Dementia‐specific excess mortality halved	$167 339	$275 902	−$108 563	$131 331	$182 034	−$50 703
Mortality doubled for people in P‐RAC	$156 752	$255 686	−$98 933	$103 685	$128 803	−$25 119
Time horizon decreased to 1‐y and difference in prob of entry to P‐RAC between int and SC arms halved	$27 783	$32 801	−$5018	$33 526	$34 031	−$505
Time horizon decreased to 3‐y and difference in prob of entry to P‐RAC between int and SC arms halved	$120 024	$147 537	−$27 513	$98 132	$108 617	−$10 485
Time horizon increased to 7‐y and difference in prob of entry to P‐RAC between int and SC arms doubled	$244 113	$420 870	−$176 756	$137 105	$217 768	−$80 663

Note

All costs AUD at 2018 prices.

Abbreviations: FIT, Frailty Intervention Trial; GTSAH, Going to Stay at Home; int, intervention; P‐RAC, permanent residential aged care; prob, probability; SC, standard care.

### 2.5 Financial implications

The number of Australians eligible to receive the GTSAH program was estimated at 31 800 people. This estimate is based on the proportion of people living with dementia in Australia with a primary co‐resident carer,^20^ projected to 2018 population numbers based on Standfield et al.^3^ It was estimated that 158 900 Australians may be suitable to receive the FIT intervention, based on the frailty prevalence in community‐dwelling Australians over 65,^21^ Australian Bureau of Statistics estimates of numbers of people aged over 65^22^ and the proportion of participants in the FIT with cognitive impairment. These estimates were also varied by 10 per cent to capture a range of plausible values.

### 2.6 Statistical analysis

Baseline characteristics and admission to P‐RAC of the FIT cognitively impaired participants were compared using chi‐square, Fisher's exact or *t* tests. Statistical significance was set at *P* < 0.05 (2‐sided).

## 3 RESULTS

### 3.1 Subgroup analysis of the multidisciplinary, multifactorial rehabilitation intervention (FIT)

There were no significant differences in age, gender, MMSE, frailty scores, use of a walking aid, grip strength and Geriatric Depression Scale (short form) score at baseline for the subgroup of participants from the FIT with cognitive impairment (MMSE 18‐24; n = 19 FIT, n = 29 SC, see Supporting Information Table S3).

The 12‐month probability of admission to P‐RAC in the cognitively impaired subgroup in the FIT was 0.03 per month (9/29 (0.31) participants) in the SC arm and 0.01 per month (2/19 (0.11) participants) in the intervention arm (*P* = 0.09). The probability of admission to high‐level care was significantly reduced in the intervention arm (0/19), in comparison with SC (6/29; *P* = 0.04). The mean total number of care days per person (high‐ and low‐level combined) was threefold lower with FIT but did not significantly differ between groups (mean ± SD days SC 54 ± 99 vs FIT 15 ± 64, *P* = 0.14). The total number of high‐level care days per person showed a borderline reduction with the FIT intervention (mean ± SD days SC 6 ± 80 days, FIT 0 ± 0 days, *P* = 0.06), but the number of low‐level care days per person did not differ significantly between study arms (mean ± SD days SC 18 ± 69 vs FIT 16 ± 64, *P* = 0.91).

### 3.2 Modelling of longer‐term costs of programs of community support for older people

**FIGURE 2:**
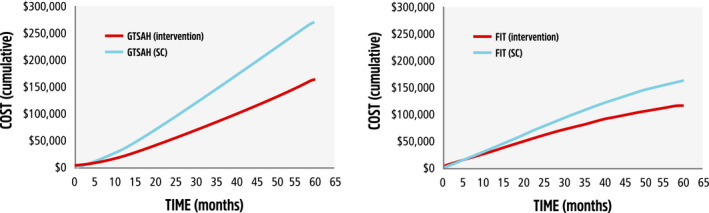
Comparison of total cumulative cost (2018 $AUD) of (A) Going to Stay at Home (GTSAH) or (B) Frailty Intervention Trial (FIT) interventions vs standard care (SC) over 5 y (discounting applied). (A) —, GTSAH (intervention); ‐ ‐ ‐, GTSAH (SC). (B) —, FIT (intervention); ‐ ‐ ‐, FIT (SC)

The model predicted that the costs of the programs would break even in approximately 5 months for GTSAH and 7 months for FIT, when compared with SC, after which the interventions were predicted to save funds (Figure 2).

**FIGURE 3:**
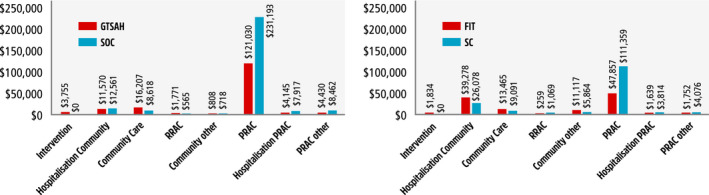
Total cost of (A) Going to Stay at Home (GTSAH) or (B) Frailty Intervention Trial (FIT) interventions vs standard care, by resource type, over 5 y (discounting applied). Abbreviations: c, cost; FIT, Frailty Intervention Trial; GTSAH, Going to Stay at Home; P‐RAC, permanent residential aged care; R‐RAC, respite residential aged care; SC, standard care; SOC, standard of care is missing. (A) 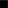, GTSAH; 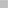, SOC. (B) 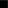, FIT; 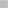, SC

The total costs accrued over the 5‐year model period, by resource type and model arm, are presented in Figure 3. For both models, the majority of costs were for P‐RAC. These costs were also the primary drivers of incremental cost savings for both interventions, driven by the predicted ability to slow the transitioning of program recipients from the community into P‐RAC. The cost of P‐RAC over 5 years was $121 030 with GTSAH compared to $231 193 with SC; and $47 857 for FIT compared to $111 359 with SC.

None of the sensitivity analyses conducted resulted in the intervention being more costly than the comparator (Table 1). These sensitivity analyses demonstrated that even if the benefits in terms of delayed admission to P‐RAC were halved, the programs still potentially saved costs over 5 years. Similarly, even if the benefits of the programs did not extend beyond the 12‐month follow‐up in the studies, the programs still provided savings. The greatest impact on the predicted cost savings occurred when the time horizon for the model was shortened, the cost of P‐RAC was varied, or when the probability of the cohort being admitted to P‐RAC was changed.

### 3.3 Potential financial implications

If the Australian population eligible for the GTSAH was within 10% of the estimated 31 800, the intervention would cost between $AUD107 to 131 million, with an associated saving of $AUD322 to 394 million at 12 months, or $AUD3 041 to 3721 million at 5 years (Table 2).

**TABLE 2:** Estimated total financial implications of providing programs delaying permanent residential care (P‐RAC) admission to people with cognitive impairment or dementia living in the community

Program	Eligible population estimate	Intervention cost (million)	12‐mo $AUD net savings (million)	P‐RAC delayed at 12 mo	Estimated 5‐y $AUD net savings (million)	P‐RAC admissions delayed at 5 y
GTSAH^a^	31 800	119	358	8402	3381	10 313
	35 000	131	394	9248	3721	11 350
	28 600	107	322	7557	3041	9275
FIT^b^	158 900	291	579	29 374	7015	50 684
	174 800	320	637	32 313	7717	55 756
	143 000	262	521	26 435	6313	45 612

^a^ Based on AIHW 2012,^20^ proportion of people with dementia with a primary resident co‐carer, projected to 2018 based on Standfield et al.^3^

^b^ Based on frailty prevalence in those community dwelling over 65 in Australia,^21^ ABS estimates of numbers of people aged over 65^22^ and the proportion in the FIT with cognitive impairment.

If the Australian population eligible for the FIT intervention was within 10% of the estimated 158 900, the intervention would cost between $AUD262 to 320 million, with an associated saving of $AUD521 to 637 million at 12 months, or $AUD6313 to 7717 at 5 years (Table 2).

## 4 DISCUSSION

This modelling predicted that investment in programs with benefits for older people living in the community with cognitive impairment could produce significant cost‐offsets and potentially be cost saving overall. The modelled cost saving was primarily driven by delayed admission to P‐RAC. There is some uncertainty in the underlying data used as they are from an observational comparison (GTSAH) and a small subgroup analysis of an RCT (FIT). In the FIT subgroup, reductions in admission to P‐RAC were statistically significant for high‐level care only. Reductions in admission to P‐RAC for combined high‐ and low‐level care were not statistically significant. Nevertheless, potential cost savings were observed in all sensitivity analyses, including analyses halving any effect on P‐RAC admissions, not extending the study effects beyond the study period (12 months), and combining both scenarios. The modelling thus indicated that investment in further evaluation of these programs is relatively low risk.

In 2017‐2018, Australian governments spent approximately $18 billion on aged care, approximately two‐thirds of this on RAC.^23^ Five billion was spent on home care and support. However, it is not clear how much of this expenditure is on evidence‐based supports. Investing a small proportion of these funds on further trials of the dyad support and multidisciplinary frailty programs, as well as similar new programs, could provide evidence of cost savings over time as well as benefits for older people in terms of staying at home for longer.

Delaying P‐RAC admission was the focus of a recent Productivity Commission review examining interventions to support carers of people with dementia.^24^ This report found that different carer support interventions were too diverse to allow pooling of results with meta‐analysis. However, results were nevertheless summarised by categories of interventions and conclusions were drawn based on these categories.^24^ We have demonstrated that conducting analyses on individual programs with some evidence of effectiveness can show potential long‐term financial benefits (in addition to patient and carer benefits).

This analysis projects the implications of providing services to a population similar to the study populations but programs may be applicable to broader populations. GTSAH was provided to people living with dementia who had undergone assessment by an aged care assessment team (ACAT) to establish eligibility for residential respite care. It is possible that the program delivered earlier in the disease course may provide greater benefits and therefore financial gains. The early iteration of this program, delivered in hospital with 12‐month ongoing support, was provided to people at an earlier point in their disease progression and demonstrated delayed admission to P‐RAC over 8 years postintervention.^11^ Also, the GTSAH study is based on a comparison to standard respite, but in fact only a proportion of community‐dwelling older people utilise respite.^25^ Similar programs have been developed to support dyads of people living with other chronic diseases in the community.^26^, ^27^, ^28^ While evidence of capacity to delay admission to P‐RAC is missing for some of these programs, other benefits such as improved carer competence, coping, family functioning and symptom burden have been observed. Thus, positive financial and health outcomes are possible.

A strength of the current analysis is that it captures a broad range of costs associated with living in the community. However, there are also some limitations in terms of applying the study and trial results to the general population. The mortality rates used in the models were based on data from a cohort with incident dementia which may differ somewhat to the cohorts modelled, and rates may vary following admission to P‐RAC. However, mortality was not a key driver of the model. A further limitation is that the data on delayed admission to P‐RAC from GTSAH were from an observational comparison, so there may be differences between the populations in the study arms. The subgroup analysis of the cognitively impaired participants from the FIT is underpowered, and reductions in P‐RAC admissions across both high‐ and low‐level care were not significant, so there is some uncertainty in these data. Also, while there were no statistically significant differences in baseline characteristics, the FIT participants were slightly younger and fewer used a walking aid, so the impact of any imbalance in baseline characteristics is uncertain (Supporting Information Table S3). Some model inputs are derived from old data (ie, from Brodaty and Peters^12^). Although system changes over time may affect these parameters, hospitalisation and community‐based costs had very little impact on the model costs (Figure 3, Supporting Information Table S5).

## 5 CONCLUSIONS

Services with preliminary evidence of benefit for older people living in the community with cognitive impairment that may delay admission to P‐RAC warrant further investment. Further trials and evaluation of such programs may provide evidence for approaches with significant financial benefits to government as well as benefits for older people living in the community. Confirmation of these modelled findings in further, larger studies and broader populations is required.

## CONFLICTS OF INTEREST

MG was employed by HammondCare who delivers the GTSAH program. MG and HB are authors on manuscripts on the GTSAH program. NF and IDC are authors of the FIT. There are no other conflicts of interest to declare.

## Supporting information

Supplementary Material
