# Supplementary material for: Supporting community‐dwelling older people with cognitive impairment to stay at home: A modelled cost analysis
Source: Australas J Ageing. 2020 Jul 1;39(4):e506–14. doi: 10.1111/ajag.12818 (PMC7818109; doi:10.1111/ajag.12818)
Supplement: Supplementary file 1 — Supplementary Material [file AJAG-39-e506-s001.pdf]

Supporting Information

Table S1 Base case input parameters dyad residential support program (GTSAH) model versus standard care (Model 1)

Parameter description	Value	Source
Costs		
Intervention arm		
Total cost of GTSAH intervention per dyad	$3,755	Personal communication, M Gresham, HammondCare, 2018 (default: in house 4-dyads participating)
Other costs 1 community intervention (per month)	$20.05	Frequency resource use: Brodaty 1991. [1] Cost: General practitioner visits; MBS Item number 23 (2018)
Other costs 2 community intervention (per month)	$4.67	Frequency resource use: Brodaty 1991. [1] Cost: Non-medical health practitioners; MBS Item Allied Health visit (2018)
Total 'Other' costs community intervention (per month)	$24.72	
Other costs 1 P-RAC intervention (per month)	$263.51	Gnanamanickam 2018.[2] Other costs = Total Health Care utilisation costs - hospital costs
Total 'Other' costs P-RAC intervention (per month)	$263.51	
SC arm		
Cost of 'intervention' in SC arm per dyad	$0.00	By definition
Other costs 1 community SC (per month)	$31.96	Frequency resource use: Brodaty 1991. [1] Cost: General practitioner visits (MRP); MBS Item number 23 (2018)
Other costs 2 community SC (per month)	$9.34	Frequency resource use: Brodaty 1991. [1] Cost: Non-medical health practitioners; MBS Item Allied Health visit(2018)
Total 'Other' costs community SC (per month)	$41.30	
Other costs 1 P-RAC SC (per month)	$263.51	Gnanamanickam 2018. [2] Other costs = Total Health Care utilisation costs - hospital costs
Total 'Other' costs P-RAC SC (per month)	$263.51	
Inputs common to both arms		
Cost of hospitalisation from community (per day)	$885	AIHW 2013. Dementia care in hospitals: costs and strategies. Cat. no. AGE 72. Canberra [3]

Parameter description	Value	Source
Cost of hospitalisation from P-RAC (per month)	$247	Gnanamanickam 2018. Hospital admissions [2]
Cost of HCP, per person with dementia (per day)	$80	Standfield 2018 [4]
Cost of respite residential aged care (per day)	$260	Gnanamanickam 2018. [2] Residential care costs + 10% for additional respite administration
Cost of permanent residential aged care for person with dementia (average across high or low care) (per day)	$237	Gnanamanickam 2018. [2] Residential care costs
Cost deflators	Various by year	AIHW 2015 Government final consumption expenditure on hospitals and nursing homes [5]
Discount rate for costs (per annum)	5%	MSAC and PBAC [6]
Risk estimates		
Inputs common to both arms		
Relative risk of mortality male with dementia vs male without dementia	2.20	Knopman 2003 [7] (dementia overall, male)
Relative risk of mortality female with dementia vs female without dementia	1.70	Knopman 2003 [7] (dementia overall, female)
Temporal inputs		
Intervention arm		
Average days hospitalisation in intervention group (per month)	0.40	Nights in general hospital per month derived from Brodaty 1991 [1]
Average number of days in respite care in intervention arm (per month)	0.21	Nights in nursing home over 12-months Brodaty 1991 [1]
SC arm		
Average days hospitalisation in SC group (per month)	0.82	Nights in general hospital per month derived from Brodaty 1991 (MRP) [1]
Average number of days in respite care in SC arm (per month)	0.13	Nights in nursing home over 12-months Brodaty 1991 (MRP) [1]
Probabilities		
Intervention arm		
Probability of entry into P-RAC (high or low care) in intervention arm (per month)	0.02	Gresham 2018 [8]
SC arm		
Probability of entry into P-RAC (high or low care) in SC arm (per month)	0.05	Gresham 2018 [8] Conservative control
Inputs common to both arms		
Probability of receiving a home care package	0.20	Personal communication S Harrison, ROSA 2013-2014 people living in community with dementia receiving home care packages
Baseline mortality	Various by age and gender	ABS life tables Australia 2014–2016 [9]
Demographic values		

Parameter description	Value	Source
Inputs common to both arms		
Proportion of cohort entering model who are females	0.48	Brodaty 1991 [1]
Mean age of males entering model (years)	70.20	Brodaty 1991 [1]
Mean age of females entering model (years)	70.20	Brodaty 1991 [1]
Weighted age across genders (calculated)	70.20	Calculated

NB. All costs presented in AUD adjusted to 2018 prices using the Government final consumption expenditure on hospitals and nursing homes index. Rounding has been applied.

Abbreviations: ABS, Australian Bureau of Statistics; GTSAH, going to stay at home programme; HCP, Home Care Package; MBS, Medicare Benefits Schedule; MRP, Memory Retraining Programme; MSAC, Medicare Services Advisory Committee; PBAC, Pharmaceutical Benefits Advisory Committee; P-RAC, Permanent residential aged care; ROSA, Registry of Older South Australians; SC, standard care.

Table S2 Base case input parameters FIT model versus SC (Model 2)

Parameter description	Value	Source
Costs		
Inputs common to both arms		
Cost of hospitalisation from P-RAC (per month)	$246.54	Gnanamanickam 2018. Hospital admissions [2]
Cost of HCP, per person with dementia (per day)	$79.97	Standfield 2018 [4]
Cost of respite residential aged care (per day)	$260.17	Gnanamanickam 2018. Residential care costs + 10% for additional respite administration [2]
Cost of permanent residential aged care for person with dementia (average across high or low care) (per day)	$236.52	Gnanamanickam 2018. Residential care costs [2]
Cost deflators	Various by year	AIHW 2015 Government final consumption expenditure on hospitals and nursing homes [5]
Discount rate for costs (per annum)	5%	MSAC and PBAC [6]
Intervention arm		
Total cost of FIT intervention	$1,833.51	Fairhall 2015; Table 3 [10]
Cost of hospitalisation (per day)	$1,180.36	Calculated from Fairhall 18-24 MMSE subgroup analysis
Other costs 1 community intervention (per month)	$107.37	Fairhall 2015; Table 4; General practitioner visits [10]
Other costs 2 community intervention (per month)	$112.83	Fairhall 2015; Table 4; Nursing other health practitioner visits [10]
Other costs 3 community intervention (per month)	$189.23	Fairhall 2015; Table 4; Transport; home help; meal delivery combined [10]

Parameter description	Value	Source
Total 'Other' costs community intervention (per month)	$409.42	Calculated
Other costs 1 P-RAC intervention (per month)	$263.51	Gnanamanickam 2018.[2] Other costs = Total Health Care utilisation costs - hospital costs
Total 'Other' costs P-RAC intervention (per month)	$263.51	Calculated
SC arm		
Cost of 'intervention' in SC arm	$0.00	By definition
Cost of hospitalisation (per day)	$1,304.46	Calculated from Fairhall 2015 18-24 MMSE subgroup analysis
Other costs 1 community SC (per month)	$104.89	Fairhall 2015; Table 4; General practitioner visits [10]
Other costs 2 community SC (per month)	$87.09	Fairhall 2015; Table 4; Nursing other health practitioner visits [10]
Other costs 3 community SC (per month)	$127.91	Fairhall 2015; Table 4; Transport; home help; meal delivery combined [10]
Total 'Other' costs community SC (per month)	$319.89	Calculated
Other costs 1 P-RAC SC (per month)	$263.51	Gnanamanickam 2018.[2] Other costs = Total Health Care utilisation costs - hospital costs
Total 'Other' costs P-RAC SC (per month)	$263.51	
Risk estimates		
Inputs common to both arms		
Relative risk of mortality male with dementia vs male without dementia	2.20	Knopman 2003 (dementia overall, male) [7]
Relative risk of mortality female with dementia vs female without dementia	1.70	Knopman 2003 (dementia overall, female) [7]
Temporal inputs		
Intervention arm		
Average days in hospital per separation	9.54	Calculated from Fairhall 2015 [10]; 18-24 MMSE subgroup analysis
Average number of days in respite care (per month)	0.04	Calculated from Fairhall 2015 [10]; 18-24 MMSE subgroup analysis
SC arm		
Average days in hospital per separation	18.00	Calculated from Fairhall 2015 [10]; 18-24 MMSE subgroup analysis
Average number of days in respite care (per month)	0.22	Calculated from Fairhall 2015 [10]; 18-24 MMSE subgroup analysis
Probabilities		
Intervention arm		
Probability of entry into P-RAC (high or low care) in intervention arm (per month)	0.01	Calculated from Fairhall 2015 [10];18-24 MMSE subgroup analysis

Parameter description	Value	Source
Probability of hospitalisation from the community (per month)	0.13	Calculated from Fairhall 2015 [10]; 18-24 MMSE subgroup analysis
SC arm		
Probability of entry into P-RAC (high or low care) in SC arm (per month)	0.03	Calculated from Fairhall 2015 [10]; 18-24 MMSE subgroup analysis
Probability of hospitalisation from the community (per month)	0.06	Calculated from Fairhall 2015 [10]; 18-24 MMSE subgroup analysis
Inputs common to both arms		
Probability of receiving a home care package	0.20	Personal communication, ROSA 2013-2014 people living in community with dementia receiving home care packages
Baseline mortality	Various by age and gender	ABS life tables Australia 2014–2016 [9]
Demographic values		
Inputs common to both arms		
Proportion of cohort entering model who are females	0.58	Calculated from Fairhall 2015 [10]; 18-24 MMSE subgroup analysis
Mean age of females entering model (years)	86.75	Calculated from Fairhall 2015 [10]; 18-24 MMSE subgroup analysis
Mean age of males entering model (years)	86.14	Calculated from Fairhall 2015 [10]18-24 MMSE subgroup analysis
Weighted age across genders (calculated)	86.49	Calculated

NB. All costs presented in AUD adjusted to 2018 prices using the Government final consumption expenditure on hospitals and nursing homes index. Rounding has been applied.

Abbreviations: ABS, Australian Bureau of Statistics; FIT, multifactorial, interdisciplinary intervention to reduce frailty in frail older people; HCP, Home Care Package; MBS, Medicare Benefits Schedule; MSAC, Medicare Services Advisory Committee; MMSE, Mini-Mental State Examination; PBAC, Pharmaceutical Benefits Advisory Committee; P-RAC, Permanent residential aged care; ROSA, Registry of Older South Australians; SC, standard care.

Figure S1 Cumulative cost of GTSAH and SC, by resource type, over 5-years (discounting applied)

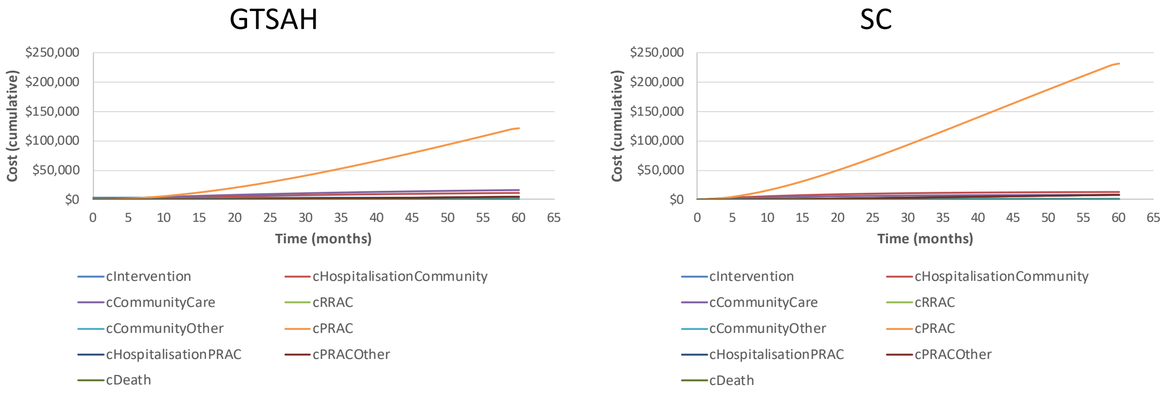

NB. All costs AUD at 2018 prices.
Abbreviations: c, cost; GTSAH, going to stay at home programme; PRAC, Permanent residential aged care; RRAC, Respite residential aged care; SC, standard care.

Table S3 Baseline characteristics for participants with cognitive impairment in FIT trial

	**Intervention**	**Control**	**P**
Total number of patients ITT (n)	19	29	
Gender, females/males (n)	11/8	17/12	0.96
Age, baseline (mean; SD)	85; 6	87; 6	0.78
MMSE score (mean; SD)	22; 2	21; 2	0.39
Frailty score (n)<br>3<br>4<br>5	 12<br>5<br>2	 17<br>9<br>3	 0.99<br>0.74<br>0.26
Walking aid at baseline (n)	4	7	0.82
Grip strength, baseline, kg (mean; SD)	15; 8	13; 7	0.69
GDS score (mean; SD)	6; 3	5; 3	0.49

Selection of programs for modelling

Members of the NHMRC cognitive-decline partnership centre including academics and representatives of three aged care providers were consulted regarding suggestions for Australian community-based programs with evidence for supporting older people to remain living in the community. This was supplemented with non-systematic searches of the literature for suitable programs in mid 2018. Reviews and articles on respite [11-13], multifactorial interventions [14], falls prevention [15] and reablement [16] were also examined for suitable programs. Searches in PubMed and google were conducted using the terms Australia AND dementia AND cost (restricted to clinical trial), also for authors Srikanth V [au], Byles J [au] Low LF [au], Jeon YH [au] AND dementia.

Criteria for programs considered suitable for modelling were those that

- were Australian, or had been implemented in Australia
- had been implemented in a dementia/cognitively impaired population
- had a reported trial/evaluation against a control group, with evidence of effectiveness on a patient outcome measure
- reported Australian costs of the intervention and economic analysis with resource use data
- reported data on the impact of the program on admission to permanent residential aged care

Table S4 lists some specific programs examined for suitability for modelling exercise and reasons for inclusion or exclusion from modelling study. Two programs were included in the modelling study.

Table S4 Programs supporting older people living in the community considered for modelling

Program name	Comments	References
Case management (COMPAS study) (Vroomen 2016)	Two Netherlands models of case management for dementia in observational study comparing to control. Resource use data does not reflect Australian context.	[17]

Program name	Comments	References
COPE (Gitlin et al 2010)	US program, Australian implementation in progress. Lack of data on impact on admission to residential aged care.	[18-20]
FIT (Fairhall et al 2015)	Trial includes cognitively impaired subgroup and suitable resource use and cost data. Authors contacted & agreed to conduct a subgroup analysis of relevant data for those with cognitive impairment. Program included in modelling.	[10,21-23]
GTSAH (Brodaty et al 1997, Gresham 2018)	Program for people living with dementia in the community. Suitable data available, authors agreed to provide intervention costs and updated data on observational study of program as currently implemented. Program included in modelling.	[24-26,1]
HIP reablement (Lewin et al 2013)	Reablement (restorative care) program. Excludes people with dementia, costs reported as median.	[27,28]
I-HARP (2017 abstract)	Comparative data not reported, ongoing trial at time of modelling. Only web reference identified. "Within 12 months of taking part in the study participants had fewer falls, fewer hospital admissions and none had moved into residential aged care."	[29-31]
Occupational therapy home visits (Cumming et al 1999)	Includes cognitive impaired participants, mostly discharged from hospital. A single component of a multifaceted program, aiming to reduce falls, rather than an analysis of a complete program. Reports residential care costs but not admission rates.	[29,30]
Transition care	Evaluation of a funded program. Data for TC in both resi and community settings (evaluated separately). Control data not concurrent (transition data for July 2005 - June 2006 (see p6), control data from Oct 2006-March 2007 (see p49).<br>Data from July 2006- Sept 2007 indicated "several jurisdictions were excluding people with dementia" (p86).	[32,33]
STICCS (Short Term Intensive Community Care and Support Service)	2005 evaluation of community care program providing "time-limited but intensive support to people who have been in hospital and have dementia or a level of confusion". No comparison data on control program.	[34]
Central Sydney Tai Chi trial (Voukelatos 2007, Haas 2006, CHERE working paper)	Tai Chi falls prevention trial. "relatively health community-dwelling people >60 years", dementia excluded. Abstract of HE analysis reports health service use costs, unclear if data available on residential aged care admissions. A single component of a multifaceted program (exercise), rather than a complete program.	[35,36]

GTSAH resource inputs

For the GTSAH program, the only source of controlled data available for some resource use data (e.g. number of GP visits, allied health visits, number of days in hospital, as per Table S1) was the Brodaty 1991 trial. Unit costs applied for these resources were from contemporary sources (i.e. 2018 prices). Whilst system changes over time may affect these parameters, removal of these inputs from the analysis (making these parameters equivalent for both arms of the model) did not change the break-even time (it remained at 5 months) and altered the incremental cost by less than 1% (see Table S5). This indicated that these values had very little impact on the model results.

Table S5 Total cumulative cost (2018 $AUD) of Going to Stay at Home (GTSAH) intervention versus standard care (SC): Univariate sensitivity analysis removing Brodaty 1991 resource estimates

Analysis description	Modelled GTSAH cohort costs		
	Intervention arm	SC arm	Incremental Cost
**Base case**	**$163,716**	**$270,035**	**-$106,319**
Brodaty 1991 resource use estimates removed from analysis	$149,567	$256,191	-$106,625

References:

1. Brodaty H, Peters KE. Cost effectiveness of a training program for dementia carers. *Int Psychogeriatr.* 1991;3(1):11-22.
2. Gnanamanickam ES, Dyer SM, Milte R et al. Direct health and residential care costs of people living with dementia in Australian residential aged care. *Int J Geriatr Psychiatry.* 2018;33(7):859-866. https://doi.org/10.1002/gps.4842.
3. Australian institute of Health and Welfare. Dementia care in hospitals costs and strategies. Cat. no. AGE 72. 2013. https://www.aihw.gov.au/getmedia/9c7deaeb-1b8c-4e40-8763-cc01560642cc/14347_20130502.pdf.aspx.
4. Standfield LB, Comans T, Scuffham P. A simulation of dementia epidemiology and resource use in Australia. *Aust N Z J Public Health.* 2017;42(3):291-295. https://doi.org/10.1111/1753-6405.12700.
5. Australian Institute of Health and Welfare. Health expenditure Australia 2015–16. Cat. no. HWE 68. 2017. https://apo.org.au/sites/default/files/resource-files/2017/10/apo-nid113061-1185756.pdf.
6. Australia Goverment. Department of Health. Manual of resource items and their associated unit costs: For use in submissions to the Pharmaceutical Benefits Advisory Committee involving economic analyses. 2016. http://www.pbs.gov.au/info/industry/useful-resources/manual.
7. Knopman DS, Rocca WA, Cha RH, Edland SD, Kokmen E. Survival study of vascular dementia in Rochester, Minnesota. *Arch Neurol.* 2003;60(1):85-90.

8. Gresham M, Heffernan M, Brodaty H. The Going to Stay at Home program: combining dementia caregiver training and residential respite care. *Int Psychogeriatr.* 2018;30(11):1697-1706. https://doi.org/10.1017/s1041610218000686.
9. Australian Bureau of Statistics. Life Tables, States, Territories and Australia, 2014-2016. 2017. https://www.abs.gov.au/AUSSTATS/abs@.nsf/allprimarymainfeatures/0A6198ABEE2E8A66CA25833500134EFB?opendocument. Accessed March 12, 2020.
10. Fairhall N, Sherrington C, Kurrle SE et al. Economic evaluation of a multifactorial, interdisciplinary intervention versus usual care to reduce frailty in frail older people. *J Am Med Dir Assoc.* 2015;16(1):41-48. https://doi.org/10.1016/j.jamda.2014.07.006.
11. Mason A, Weatherly H, Spilsbury K et al. A systematic review of the effectiveness and cost-effectiveness of different models of community-based respite care for frail older people and their carers. *Health Technol Assess.* 2007;11(15):1-157, iii. https://doi.org/10.3310/hta11150.
12. Dementia Australia. Aged Care Financing Authority (ACFA) Respite Care Consultation Submission from Dementia Australia. 2018. https://www.dementia.org.au/files/submissions/DA-submission-ACFA-Respite-Care-Consultation.pdf. Accessed March 12, 2020.
13. Maayan N, Soares-Weiser K, Lee H. Respite care for people with dementia and their carers. *Cochrane Database Syst Rev.* 2014;(1):Cd004396. https://doi.org/10.1002/14651858.CD004396.pub3.
14. Hopewell S, Adedire O, Copsey BJ et al. Multifactorial and multiple component interventions for preventing falls in older people living in the community. *Cochrane Database Syst Rev.* 2018;7:Cd012221. https://doi.org/10.1002/14651858.CD012221.pub2.
15. Gillespie LD, Robertson MC, Gillespie WJ et al. Interventions for preventing falls in older people living in the community. *Cochrane Database Syst Rev.* 2012;(9):Cd007146. https://doi.org/10.1002/14651858.CD007146.pub3.
16. Poulos CJ, Bayer A, Beaupre L et al. A comprehensive approach to reablement in dementia. *Alzheimers Dement (N Y).* 2017;3(3):450-458. https://doi.org/10.1016/j.trci.2017.06.005.
17. MacNeil Vroomen J, Bosmans JE, Eekhout I et al. The Cost-Effectiveness of Two Forms of Case Management Compared to a Control Group for Persons with Dementia and Their Informal Caregivers from a Societal Perspective. *PLoS One.* 2016;11(9):e0160908. https://doi.org/10.1371/journal.pone.0160908.
18. Clemson L, Laver K, Jeon YH et al. Implementation of an evidence-based intervention to improve the wellbeing of people with dementia and their carers: study protocol for 'Care of People with dementia in their Environments (COPE)' in the Australian context. *BMC Geriatr.* 2018;18(1):108. https://doi.org/10.1186/s12877-018-0790-7.
19. Fortinsky RH, Gitlin LN, Pizzi LT et al. Translation of the Care of Persons with Dementia in their Environments (COPE) intervention in a publicly-funded home care context: Rationale and research design. *Contemp Clin Trials.* 2016;49:155-165. https://doi.org/10.1016/j.cct.2016.07.006.
20. Gitlin LN, Winter L, Dennis MP, Hodgson N, Hauck WW. A biobehavioral home-based intervention and the well-being of patients with dementia and their caregivers: the COPE randomized trial. *Jama.* 2010;304(9):983-991. https://doi.org/10.1001/jama.2010.1253.
21. Fairhall N, Aggar C, Kurrle SE et al. Frailty Intervention Trial (FIT). *BMC Geriatr.* 2008;8:27. https://doi.org/10.1186/1471-2318-8-27.
22. Fairhall N, Sherrington C, Cameron ID et al. A multifactorial intervention for frail older people is more than twice as effective among those who are compliant: complier average causal effect analysis of a randomised trial. *J Physiother.* 2017;63(1):40-44. https://doi.org/10.1016/j.jphys.2016.11.007.
23. Fairhall N, Sherrington C, Kurrle SE, Lord SR, Lockwood K, Cameron ID. Effect of a multifactorial interdisciplinary intervention on mobility-related disability in frail older people: randomised controlled trial. *BMC Med.* 2012;10:120. https://doi.org/10.1186/1741-7015-10-120.
24. Brodaty H, Gresham M. Effect of a training programme to reduce stress in carers of patients with dementia. *BMJ.* 1989;299(6712):1375-1379.

25. Brodaty H, Gresham M, Luscombe G. The Prince Henry Hospital dementia caregivers' training programme. *Int J Geriatr Psychiatry.* 1997;12(2):183-192.
26. Brodaty H, McGilchrist C, Harris L, Peters KE. Time until institutionalization and death in patients with dementia. Role of caregiver training and risk factors. *Arch Neurol.* 1993;50(6):643-650.
27. Lewin G, Concanen K, Youens D. The Home Independence Program with non-health professionals as care managers: an evaluation. *Clin Interv Aging.* 2016;11:807-817. https://doi.org/10.2147/cia.s106180.
28. Lewin GF, Alfonso HS, Alan JJ. Evidence for the long term cost effectiveness of home care reablement programs. *Clin Interv Aging.* 2013;8:1273-1281. https://doi.org/10.2147/cia.s49164.
29. Cumming RG, Thomas M, Szonyi G et al. Home visits by an occupational therapist for assessment and modification of environmental hazards: a randomized trial of falls prevention. *J Am Geriatr Soc.* 1999;47(12):1397-1402. https://doi.org/10.1111/j.1532-5415.1999.tb01556.x.
30. Salkeld G, Cumming RG, O'Neill E, Thomas M, Szonyi G, Westbury C. The cost effectiveness of a home hazard reduction program to reduce falls among older persons. *Aust N Z J Public Health.* 2000;24(3):265-271. https://doi.org/10.1111/j.1467-842x.2000.tb01566.x.
31. The University of Sydney. Interdisciplinary Home-bAsed Reablement Program (I-HARP). https://www.sydney.edu.au/medicine-health/our-research/research-centres/i-harp.html. Accessed March 10, 2020.
32. Comans TA, Peel NM, Cameron ID, Gray L, Scuffham PA. Healthcare resource use in patients of the Australian Transition Care Program. *Aust Health Rev.* 2015;39(4):411-416. https://doi.org/10.1071/ah14054.
33. Transition Care Working Group. National Evaluation of the Transition Care Program RFT 206/0506 Final Evaluation Report 31 May 2008. 2008. https://pdfs.semanticscholar.org/b425/e28ede16de41be72641c481cec1d99cda3b8.pdf. Accessed March 12, 2020.
34. Elton Consulting. *Evaluation of the Short Term Intensive Community Care and Support Service. Final report prepared for The Hammond Care Group by Elton Consulting*. Elton Consulting; 2005.
35. Voukelatos A, Cumming RG, Lord SR, Rissel C. A randomized, controlled trial of tai chi for the prevention of falls: the Central Sydney tai chi trial. *J Am Geriatr Soc.* 2007;55(8):1185-1191. https://doi.org/10.1111/j.1532-5415.2007.01244.x.
36. Haas M. Economic analysis of Tai Chi as a means of preventing falls and falls related injuries among older adults, CHERE Working Paper 2006/4. 2006. https://ideas.repec.org/p/her/chewps/2006-4.html.
